# Massive Spread of OXA-48 Carbapenemase-Producing Enterobacteriaceae in the Environment of a Swiss Companion Animal Clinic

**DOI:** 10.3390/antibiotics11020213

**Published:** 2022-02-08

**Authors:** Kira Schmitt, Michael Biggel, Roger Stephan, Barbara Willi

**Affiliations:** 1 Institute for Food Safety and Hygiene, University of Zurich, CH-8057 Zurich, Switzerland; kira.schmitt@uzh.ch (K.S.); michael.biggel@uzh.ch (M.B.); 2 Graduate School for Cellular and Biomedical Sciences, University of Bern, CH-3012 Bern, Switzerland; 3 Clinic for Small Animal Internal Medicine, University of Zurich, CH-8057 Zurich, Switzerland; bwilli@vetclinics.uzh.ch

**Keywords:** CPE, CTX-M15, ESBL, IPC, hand hygiene, carbapenemase, OXA-48, IncL, plasmid

## Abstract

Background: Companion animal clinics contribute to the spread of antimicrobial resistant microorganisms (ARM) and outbreaks with ARM of public health concern have been described. Methods: As part of a project to assess infection prevention and control (IPC) standards in companion animal clinics in Switzerland, a total of 200 swabs from surfaces and 20 hand swabs from employees were collected during four days in a medium-sized clinic and analyzed for extended spectrum beta-lactamase-producing Enterobacteriaceae (ESBL-E), carbapenemase-producing Enterobacteriaceae (CPE), vancomycin-resistant enterococci (VRE), and methicillin-resistant staphylococci (MRS). Results: A total of 22 (11.0%) environmental specimen yielded CPE, 14 (7.0%) ESBL-E, and 7 (3.5%) MRS; MR *Staphylococcus aureus* were isolated from two (10.0%) hand swabs. The CPE isolates comprised *Escherichia coli*, *Klebsiella pneumoniae*, *Enterobacter hormaechei*, *Citrobacter braakii*, and *Serratia marcescens*. Whole genome sequencing revealed that all CPE carried closely related *bla*_OXA-48_ plasmids, suggesting a plasmidic spread within the clinic. The clinic exhibited major deficits in surface disinfection, hand hygiene infrastructure, and hand hygiene compliance. CPE were present in various areas, including those without patient contact. The study documented plasmidic dissemination of *bla*_OXA-48_ in a companion animal clinic with low IPC standards. This poses a worrisome threat to public health and highlights the need to foster IPC standards in veterinary clinics to prevent the spread of ARM into the community.

## 1. Introduction

Antimicrobial resistance has been declared as one of “the greatest and most urgent global risks” by the United Nations General Assembly [1]. Antimicrobial resistant microorganisms (ARM) are estimated to cause the death of over 700,000 people per year and pose a threat to the healthcare system [2]. Carbapenemase-producing Enterobacteriaceae (CPE) represent ARM of special concern due to their ability to hydrolyze carbapenems. The World Health Organization (WHO, Geneva, Switzerland) classifies carbapenem antibiotics as critically important for human health and considers them as antimicrobials of last resort due to their broad spectrum of activity against several gram-positive and gram-negative bacteria [3]. Carbapenem resistance encoded by *bla*_OXA-48_ is often located on transferable L/M complex plasmids. These plasmids can possess derepressed transfer properties, allowing them to efficiently spread horizontally [4]. CPE have caused outbreaks and hospital-acquired infections in human healthcare worldwide [5,6,7,8,9,10,11]. In companion animal clinics, CPE have, so far, only scarcely been reported [12,13,14,15]. Of note, the use of carbapenems in companion animal medicine is restricted to special indications and has not been widely reported [16]. Companion animal clinics harbor multiple factors that foster the selection of antimicrobial resistant microorganisms (ARM): a high number of hospitalized and debilitated patients, a high percentage of animals receiving antimicrobial therapy, and daily invasive procedures associated with numerous hand-patient contacts.

As part of an action plan to combat the spread of ARM in companion animal clinics, a project to evaluate the effect of infection prevention and control (IPC) implementation in five veterinary clinics on environmental contamination with ARM, hand hygiene, and IPC standards was launched in Switzerland. During this project, a spread of CPE and extended-spectrum beta-lactamase producing Enterobacteriaceae (ESBL-E) was identified in one of the clinics which showed extensive environmental contamination. The aims of the present study were to characterize the isolates, to investigate potential transmission events, and to assess IPC standards in the affected institution.

## 2. Results

### 2.1. IPC Standards and Hand Hygiene Adherence

The dissemination of resistant bacteria was identified in a medium-sized clinic in Switzerland that comprised 45 staff members, offered a 24/7 emergency service and an intensive care unit (ICU), and treated around 13,000 ambulatory and stationary dogs and cats per year. In the IPC standard assessment the clinic reached 59 out of 102 (58%) total scoring points (Table 1). The IPC audit revealed major deficits regarding cleaning and disinfection, hand hygiene infrastructure, isolation measures, equipment in examination rooms, and antimicrobial use. Written protocols on cleaning and disinfection were not implemented. Hand washing stations and hand disinfection dispensers were not available in all areas where patients were treated. The clinic had limited IPC management in place and staff education regarding IPC was scarce.

Overall, 525 hand hygiene observations were collected in the clinic. Overall hand hygiene compliance was 14.9% (95% CI 12.1–18.2%). Significant differences were observed between clinical areas (*p* = 0.007) and hand hygiene indications (*p* < 0.0001). The highest hand hygiene compliance was found in the wards (23.9%, 95% CI 17.5–31.8%), followed by the consultation area (13.4%, 95% CI 8.5–20.4%), the ICU (11.8%, 95% CI 7.3–18.6%), and the pre-operation preparation area (10.2%, 95% CI 6.2–16.4%). Hand hygiene was more commonly performed after body fluid exposure risk (19.0%, 95% CI 12.9–27.0%), after patient contact (16.5%, 95% CI 11.2–23.8%), and after touching the patient’s surrounding (18.2%, 95% CI 11.8–26.9%), than before patient contact (9.7%, 95% CI 5.5–16.6%) and before clean/aseptic/invasive procedures (7.8%, 95% CI 3.4–17.0%).

### 2.2. Environmental Swabs and Hand Swabs

Overall, 200 environmental swabs and 20 hand swabs were collected and analyzed. From the environmental specimens, 31 (15.5%) tested positive for at least one of the investigated ARM. CPE were detected in 22 (11.0%, 95% CI 7.4–16.1) specimens, ESBL-E in 14 (7.0%, 95% CI 4.2–11.4), and methicillin-resistant staphylococci (MRS) in 7 (3.5%, 95% CI 1.7–7.0) specimens. MR *Staphylococcus aureus* were isolated from two hand swabs (10.0%, 95% CI 1.77–30.1). Vancomycin-resistant enterococci (VRE) were not detected in any specimens.

**Figure 1:**
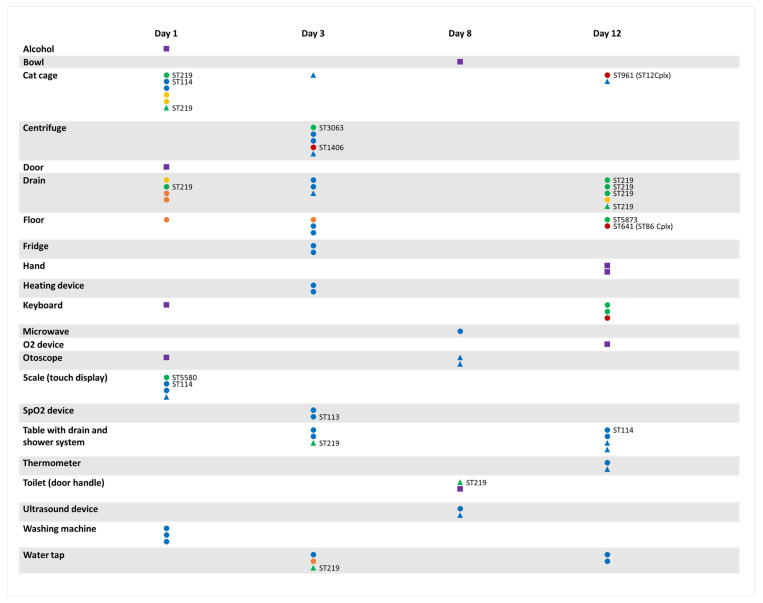
Timeline of CPE, ESBL-E, and MRS isolated from the clinical environment. Each column refers to one sampling day. Each horizontal line refers to a specimen obtained from the same environmental surface over time. Isolates of the same surface on the same day derived from subcultures of the same samples. Negative test results are omitted. The sequence types are indicated at the right side of the symbols for the *Escherichia coli*, *Klebsiella pneumoniae*, and the *Enterobacter hormaechei* isolates for which whole genome sequencing was conducted. Circles indicate CPE, triangles indicate ESBL-E, and squares indicate MRS. The color of the symbols indicate: purple, *Staphylococcus* spp.; orange, *Serratia maracescens*; yellow, *Citrobacter braakii*; blue, *Enterobacter hormaechei*; red, *Escherichia coli*; and green, *Klebsiella pneumoniae.* Abbreviations: ST, sequence type; CPE, carbapenemase-producing Enterobacteriaceae; ESBL-E, extended spectrum beta-lactamase-producing Enterobacteriaceae; and MRS, methicillin-resistant staphylococci.

The environmental isolates originated from 22 different surfaces from all areas across the clinic, including those where no animals were permitted, such as the staff kitchen, the toilet, and the laboratory (Figure 1). The percentage of ARM positive samples from the environment per day ranged from 10.0% (95% CI 4.3–21.4, day 3) to 18.0% (95% CI 9.8–30.8, days 1, 2 and 4). The isolates comprised several species and sequence types. The ESBL-E and CPE isolates included *Escherichia coli* ST961, ST12, ST641, and ST1406; *Klebsiella pneumoniae* ST219, ST3063, ST857, ST5580, and ST5873; *Enterobacter hormaechei*; *Citrobacter braakii*; and *Serratia marcescens* (Figure 1, Table S1). All CPE carried *bla*_OXA-48_; the ESBL-E carried *bla*_CTX-M-15_ and/ or *bla*_SHV-12_; the MRS isolates all harbored the *mecA* gene (Table S1).

**Figure 2:**
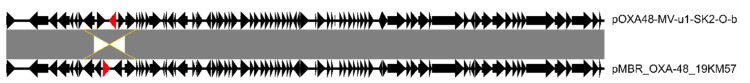
Genetic similarity of *bla*_OXA-48_-harboring plasmids from this and a previous study. Comparison of pOXA48-MV-u1-SK2-O-b (CP085868.1) from *Klebsiella pneumoniae* ST219 isolate OXA48-MV-u1-SK2-O and the publicly available sequence of pMBR_OXA-48_19KM57 (CP039950.1) from *Klebsiella pneumoniae* ST11 isolate 19KM57, which was found in a different veterinary clinic in Switzerland [19]. Shaded boxes between sequences indicate homologous regions (100% sequence identity). The *bla*_OXA-48_ gene (colored in red) was part of a 5.6 kb region (box outlined in yellow) flanked by *IS*10A insertion elements. This region was inverted in pOXA48-MV-u1-SK2-O-b when compared to pMBR_OXA-48_19KM57. The figure was generated with Easyfig 2.1 [21].

To investigate a potential clonal dissemination of isolates and horizontal spread of *bla*_OXA-48_-encoding plasmids within the clinical environment, 12 *bla*_OXA-48_ harboring isolates (1 *C. braakii*, 4 *Enterobacter* spp., 2 *E. coli* (ST961 and ST1406), 4 *K. pneumoniae*, (2 ST219, ST3063, and ST5873) and 1 *S. marcescens*) were subjected to whole genome sequencing (WGS) (Table S2). The four *Enterobacter* spp. isolates were identified as *E. hormaechei* according rMLST analyses and assigned to ST113 and ST114 (3x) using the *Enterobacter cloacae* complex MLST scheme. *E. cloacae* complex clades harboring ST113 and ST114 isolates were previously designated as *E. hormaechei* subsp. steigerwaltii, and *E. hormaechei* subsp. xiangfangensis (or *E. xiangfangensis*), respectively [17,18]. Hybrid assemblies of three isolates (2 *K. pneumoniae* (ST219 and ST5873), and 1 *S. marcescens*) which additionally underwent long-read sequencing revealed the presence of identical *bla*_OXA-48_-carrying 63,589 bp IncL plasmids in each isolate. Read-mapping-based approaches confirmed the presence of this pOXA-48-like plasmid in all other sequenced CPE isolates. The plasmid was conserved across the different clones and species, deviating only in *E. coli* MV-r4-SK2-C by one SNP (T37049A). In the NCBI nucleotide collection, the identical plasmid (63,589 bp; 100% sequence identity and query cover; ignoring inversions) was found in 44 genomes from global isolates (33 *K. pneumoniae*, 8 *E. coli*, and 3 *Citrobacter freundi*i) deriving, amongst others, from humans and the environment of another Swiss veterinary clinic [19]. The plasmid obtained in the veterinary clinic differed structurally by a 5.6 kb inversion (Figure 2). The genetic structure of the plasmid was described before (pEc_MW04_OXA, [20]) and did not contain antimicrobial resistance genes other than *bla*_OXA-48_.

WGS also demonstrated a clonal spread of CPE across different sources and sampling days in the clinic environment: three of the four sequenced *E. hormaechei* belonged to ST114 and differed by ≤7 pairwise whole genome SNPs. The three isolates were found on three distinct surfaces in different areas of the clinic and collected over a period of 12 days. Similarly, the two sequenced *K. pneumoniae* ST219 isolates differed by four whole genome SNPs, suggesting a common origin. ST219 was found throughout the clinic and on every sampling day (Figure 1).

The number of antimicrobial resistance genes identified in the entire genome of the sequenced CPE isolates ranged from only one gene (*bla*_OXA-48_) in each of the two *E. coli* isolates, to 17 genes in both clones associated with spread, i.e., *E. hormaechei* ST114 and *K. pneumoniae* ST219 (Table S2). These included *bla*_CTX-M-15_ and fosA in the two *K. pneumoniae* ST117 isolates, and mcr-9 and fosA in the three *E. hormaechei* ST114 isolates, amongst others.

AmpC-type β-lactamase genes were identified in the *C. braakii* isolate (*bla*_CMY-101_), in the *E. hormaechei* ST113 isolate (*bla*_ACT-15_), in the three *E. hormaechei* ST114 isolates (*bla*_ACT-16_), and in the *S. marcescens* isolate (*bla*_SRT-2_). BLAST searches of the NCBI nucleotide collection using contigs harboring the different AmpC-type β-lactamase genes as queries suggested chromosomal locations in all six cases. Likewise, *bla*_SHV_ genes identified in the four *K. pneumoniae* isolates were located in the chromosome. Hybrid assemblies of *K. pneumoniae* MV-u1-SK2-O, *K. pneumoniae* MV-v4-SK2-O, and *S. marcescens* MV-u1-SK1-O revealed the presence of five, two, and two plasmids, respectively. The (co-)location of antimicrobial resistance genes in the chromosome or plasmids are shown in Table S3.

Resistance profiles were determined for all ESBL-E and CPE isolates collected in this study (Table S1). All ESBL-E and CPE isolates showed resistance to ampicillin and cephazolin. Most strains were resistant to cefotaxime, amoxicillin-clavulanic acid, sulfamethoxazole trimethoprim, azithromycin, streptomycin, and tetracyclin. One isolate was resistant to fosfomycin. Of the CPE, two isolates were resistant to ertapenem. One of these strains was additionally resistant to meropenem and imipenem.

## 3. Discussion

Our study documents an extensive environmental contamination with CPE, ESBL-E, and MRS isolates in a companion animal clinic in Switzerland. A total of 15.5% of the environmental specimens harbored at least one of the investigated ARM. From 11.0% of the environmental specimens, a CPE was isolated. This is already the second report of a CPE dissemination in a companion animal clinic in Switzerland. Recently, a CPE outbreak caused by *E. coli* harboring the carbapenemase gene *bla*_OXA-181_ was documented in a companion animal clinic in Switzerland. During the outbreak, 21.6% of the hospitalized dogs and cats were colonized with *E. coli bla*_OXA-181_ after two days of hospitalization, whereas only one of the tested animals was colonized at the time of admission (0.75%) [12]. Of concern, carriage of a closely related *bla*_OXA181_-positive *E. coli* was also detected in a staff member of the clinic [22]. Furthermore, CP *K. pneumoniae* (ST11, *bla*_OXA-48_) was found in 22% of the environmental sampling sites collected in the ICU and the emergency room in this institution [23]. In contrast to the aforementioned outbreak, the successful spread in our study relied not only on clonal, but also on plasmidic dissemination of the IncL plasmid. Based on SNP analyses and MLST, two dissemination clusters were detected in the present study, namely *E. hormaechei* ST114 and *K. pneumoniae* ST219. Near-identical strains were isolated from different sampling sites, indicating the presence of a common source. Identical IncL OXA-48 plasmids were found in 11 of the 12 sequenced isolates, which belonged to 5 different bacterial species. The identified IncL plasmid has been isolated world-wide from various sources, including veterinary and human healthcare settings [5,6,19,20,24]. Of note, the IncL plasmid was compositionally identical to a plasmid isolated from another Swiss veterinary referral hospital (5.6 kb inversion difference [19]). A connection between these two clinics cannot be ruled out because animal patients are frequently referred between the two institutions. The repeated occurrence of this plasmid in companion animal clinics might contribute to the spread of CPE, posing a threat to public health. 

Contamination in companion animal clinics may vary across sampling days, as has been previously reported [25]. This was also evident in the present study, where ARM contamination rates ranged from 10.0–18.0% over the 4 sampling days. In our study, ESBL-E was found in 7.0% of the 200 samples collected from the clinical environment. A recent study in five Swiss companion animal clinics found ESBL-E contamination in 0–2% of the environmental specimens [23]. ESBL-E were the most common ARM acquired by dogs and cats during hospitalization in four clinics in Switzerland [26] and were recently shown to spread to the household environment and to the animal owners after patient discharge [25]. In the latter study, transmission chains for four different high-risk human pathogenic strains of ESBL-E were documented over a 45-day observation period in a companion animal ICU. After discharge, the animal owners showed colonization with closely related ESBL-E strains and extensive contamination of the household environment with ESBL-E was documented. Transmission of CPE and ESBL-E between owners and their pets was also reported in other studies [27,28], which underlines the public health concern of ARM dissemination by companion animal veterinary institutions [25,28,29,30,31,32,33,34].

ARM-positive samples were commonly retrieved from areas with no patient contact, such as the kitchen, the laboratory, and the toilets. In a recent study conducted at a companion animal shelter, these areas were reported to have a high bacterial contamination [35]. Thus, areas with little to no patient contact in a veterinary clinic could pose a reservoir for ARM and a focus should also be placed on these locations when implementing cleaning and disinfection protocols. The conducted audit revealed a low overall IPC score in the investigated clinic (58% of the maximum IPC score). This goes in line with results from the previous study, where three companion animal clinics in Switzerland with extensive ARM contamination reached only 28–52% of the maximum IPC score [23]. Of note, hand hygiene infrastructure was absent in many patient areas in the herein described clinic, and hand hygiene compliance was worryingly low, with an overall compliance of only 14.9%. This compliance is even lower than recently reported in several companion animal institutions in Switzerland and companion animal clinics abroad, which reported a hand hygiene compliance of 14–42% [36,37,38,39,40]. Hand hygiene is regarded a key component to interrupt transmission chains for ARM and other pathogens in hospital settings [41,42,43,44]. The very low hand hygiene compliance before clean/aseptic/invasive procedures (7.8%) and before patient contact (9.7%) in this clinic poses the patients at greater risk to acquire ARM through the hands of the healthcare workers.

The present study also has its limitations. For one, the IPC scoring system is based on a consensus which might be subjective to interpretation. Additionally, animal patients and healthcare workers were not sampled. However, recent studies have already documented closely related ARM, including CPE, in patients, healthcare workers, and the clinical environment of companion animal clinics [12,22,26,45]. The number of hand swabs investigated in the present study was limited, and the prevalence of ARM might thus be over- or underestimated. 

## 4. Materials and Methods

### 4.1. Study Set-Up and IPC Evaluation

IPC standards in the clinic were evaluated by direct audit as previously described [23] and an adapted IPC audit protocol that included fifteen areas of IPC was applied [46]. The audit assessed general IPC management, staff education, cleaning/disinfection, management of waste, vector control, equipment in examination rooms, isolation measures, handling of patients with ARM, hand hygiene equipment, personal hygiene, protection of employees, protective clothing, medication, use of antimicrobials, and miscellaneous (Table S4). A scoring system (0: not fulfilled; 1: partially fulfilled; 2: completely fulfilled) was applied, and a total score calculated as described [23]. Participation in the study was voluntary and was not reimbursed. After the IPC audit, the sampling, and the hand hygiene observations were completed, the participating clinic received a written report of all results, highlighting the IPC deficits and an action plan for IPC and hand hygiene implementation.

### 4.2. Hand Hygiene Evaluation

Hand hygiene compliance was evaluated according to the WHO five moments of hand hygiene (after body fluid exposure risk, after patient contact, after touching the patient’s surroundings, before clean/aseptic/invasive procedures, and before patient contact) using the CleanHands application version February 2021 (Swissnoso, National Centre for Infection Prevention) as previously described [37]. Briefly, the CleanHands application was used to evaluate hand hygiene (i.e., carried out or not) in four different clinical areas: consultation rooms, wards, ICU, and pre-operation preparation area. Hand hygiene compliance across professional groups (veterinarians, nurses, and others, i.e., personnel not allocated to the aforementioned categories) was analyzed. According to WHO guidelines, glove usage was not categorized as a hand hygiene event. All observations were carried out by the same observer over a period of one week; the observer was previously trained by an experienced observer [36]. After digital recording, the data was extracted from the software as Excel files for further statistical analyses. Non-coded hand hygiene observations, i.e., those that could not be matched to one of the five moments of hand hygiene, were excluded from statistical analysis.

### 4.3. Statistical Analysis

The commercially available GraphPad PRISM^®^ software (San Diego, CA, USA) was used for statistical analysis. Descriptive statistics were conducted for the hand hygiene compliance (%, number of correct hand hygiene events per total number of observed hand hygiene events), and binomial confidence intervals for hand hygiene compliance were calculated using the hybrid Wilson/Brown method [47]. Contingency tables were calculated using the chi-square test. Significance was set at *p* < 0.05.

### 4.4. Microbiological Evaluation

Swab samples from a predetermined list of surfaces (Table S5) and from veterinary employees’ hands were collected on four different days over a two-week period. As previously described [37], hand swabs of the entire dominant hand palm, fingers, and thumb were collected before and after patient contact using a sterile cotton swab moisturized with 0.85% saline solution. If gloves were worn, hand swabs were taken from the gloved hand directly, before and after patient contact. To reduce potential observer bias, hand swabs were taken during busy daily procedures and in areas with a high density of patients and personnel. The healthcare workers were approached immediately before animal contact without any prior announcement. A coded sample collection procedure was used, and hence, no personal data was collected from the study participants to ensure that employees did not feel obliged to change their hand hygiene behavior. All study participants gave written informed consent, and the study protocol was approved by the Swiss Ethics Committees on research involving humans (approval no. 2019-00768).

Hand swabs were processed immediately after sample collection, as previously published [37]. The swabs were homogenized for 60 s in 10 mL peptone water (BioRad, Hercules, CA, USA) using in a Stomacher^®^ 400 (Seward, Worthing, UK). The homogenate of each sample was thereafter enriched (37 °C, 24 h), followed by selective enrichment for ESBL-E and CPE in Enterobacteriaceae enrichment broth (Oxoid, Hampshire, UK), in BHI (BioRad, Hercules, CA, USA) with 6.5% saline solution for VRE, and additionally in Mueller Hinton broth (Oxoid, Hampshire, UK) with 6.5% saline solution, followed by an enrichment in tryptone soy broth (Becton Dickinson, Allschwil, Switzerland) with 4 mg/L cefoxitin and 75 mg/L aztreonam for the detection of MRS. ESBL-E were screened by using the chromogenic medium Brilliance™ ESBL Agar (Oxoid, Hampshire, UK), CPE by using chromID^®^ CARBA SMART Bi-Plate-Agar (bioMérieux, Marcy-l’Étoile, France), VRE by using the Brilliance™ VRE Agar (Oxoid, Hampshire, UK)m and MRS by using the Brilliance™ MRSA2 Agar (Oxoid, Hampshire, UK), according to the manufacturer’s instructions. Species identification was conducted by using matrix-assisted laser desorption/ionization time-of-flight mass spectrometry (MALDI-TOF–MS, Bruker Daltronics, Bremen, Germany).

Polymerase chain reaction (PCR) was carried out to screen for the presence of genes encoding *bla*_CTX-M_ group enzymes, *bla*_SHV_, and *bla*_TEM_, as previously described [48,49,50,51]. PCR targeting *bla*_VIM_, *bla*_KPC_, *bla*_OXA-48_-like, and *bla*_NDM_ genes was carried out using custom synthesized primers (Microsynth, Balgach, Switzerland) and conditions published previously [52,53]. Multiplex PCR for the presence of *vanA*, *vanB*, and *vanC_1,2_*_,3_ was conducted as previously described using custom synthesized primers (Microsynth, Balgach, Switzerland) [54]. PCR for the presence of *mecA* and *mecC* was conducted using custom synthesized primers (Microsynth, Balgach, Switzerland), as previously described [55,56]. 

Antimicrobial susceptibility testing was carried out for all ESBL-E and CPE isolates as previously described [25]. Antimicrobial susceptibility testing was performed for Enterobacteriaceae in accordance with the Clinical and Laboratory Standards Institute (CLSI) performance standards [57] using the disk-diffusion method on Mueller Hinton plates (Oxoid, Hampshire, UK) and the 16 antibiotics: ampicillin (AM), amoxicillin with clavulanic acid (AMC), azithromycin (AZM), cefazolin (CZ), cefepime (FEP), cefotaxime (CTX), chloramphenicol (C), ciprofloxacin (CIP), fosfomycin (FOS), gentamicin (G), kanamycin (K), nalidixic acid (NA), nitrofurantoin (F/M), streptomycin (S), sulfamethoxazole trimethoprim (SXT), and tetracycline (TE) (Becton Dickinson, Allschwil, Switzerland). Results were interpreted according to CLSI standards [57]. For azithromycin, an inhibition zone of ≤12 mm was interpreted as resistant. In addition, the minimal inhibitory concentrations of the carbapenem antibiotics ertapenem, imipenem, and meropenem were determined for all CPE isolates. 

For MRS isolates, antimicrobial susceptibility profiling was performed using the automated VITEK^®^ two compact system (bioMérieux, Marcy l’Etoile, France) with the AST-GP80 susceptibility testing card (bioMérieux, Nürtingen, Germany).

### 4.5. Whole-Genome Sequencing and Genomic Analyses

A subset of 12 CPE isolates underwent Illumina short-read sequencing, of which 3 were additionally subjected to nanopore long-read sequencing (Table S2). For short-read sequencing, genomic DNA was extracted using the DNeasy Blood & Tissue Kit (Qiagen, Hilden, Germany). Libraries were prepared using the Nextera DNA Flex Library Preparation Kit (Illumina) and sequenced on the Illumina MiniSeq platform with 2 × 150 bp paired-end chemistries. Draft genomes were assembled using SPAdes v3.14.1 [58] implemented in shovill v1.1.0 (github.com/tseemann/shovill; accessed on 1 October 2021) [59]. For nanopore sequencing, genomic DNA was extracted with the MasterPure Complete DNA and RNA Purification Kit (Lucigen). Multiplex libraries were prepared using the SQK-LSK109 ligation sequencing kit with the EXP-NBD114 native barcoding expansion kit (Oxford Nanopore Technologies). Libraries were sequenced on a MinION Mk1B device using the FLO-MIN106 (R9) flow cell (Oxford Nanopore Technologies). Hybrid assemblies were produced with Unicycler 0.4.8 [60,61]. Taxonomy was assigned using rMLST [61]. Antimicrobial resistance genes and plasmid replicons were identified using abricate 1.0.1 (github.com/tseemann/abricate; accessed on 1 October 2021), in conjunction with the ResFinder [62] and PlasmidFinder [63] database, respectively. Multi-locus sequence types (MLST) were determined using mlst 2.19.0 (github.com/tseemann/mlst; accessed on 1 October 2021). Whole genome SNPs among *K. pneumoniae* ST219 and *E. hormaechei* ST114 isolates were detected from Illumina read data using the CFSAN SNP pipeline v2.2.1 [64], with assemblies of isolates MV-u1-SK2-O and MV-oo4-C as references, respectively. 

The variability of pOXA-48-like plasmids in isolates only subjected to short-read sequencing was determined by SNP detection using the CFSAN pipeline with pOXA48-MV-u1-SK2-O-b (circular plasmid from the hybrid assembly of isolate MV-u1-SK2-O, accession CP085868) as reference. Plasmid coverage and identified SNPs were confirmed by read-mapping and variant detection implemented in CLC Genomics Workbench 21.0.4.

### 4.6. Data Availability

Sequencing data and genome assemblies generated as part of this study are available under BioProject no. PRJNA774102. Genome accession numbers of all investigated isolates are listed in Table S2.

## 5. Conclusions

Our results indicate that plasmidic dissemination of *bla*_OXA-48_ in companion animal clinics may occur and poses a worrisome threat to public health. The IncL plasmid was found in five different bacterial species and isolated on each of four sampling days in a companion animal clinic in Switzerland. IPC standards, hand hygiene equipment, and hand hygiene adherence were largely insufficient in the clinic, and improvement in these aspects might support the containment of the ARM dissemination. Areas without patient contact were also commonly contaminated, and cleaning and disinfection protocols should not omit these areas, as they might represent a reservoir for ARM. The study highlights the need to develop and implement evidence-based IPC concepts and hand hygiene trainings in veterinary clinics to prevent the spread of ARM into the community. The ongoing project will analyze the effect of IPC implementation as part of an intervention strategy.

## Figures and Tables

**Table 1:** IPC areas and audit scores.

IPC Area	IPC Audit Score/Maximum IPC Score
IPC management	1/10
Staff education	5/12
Cleaning/disinfection	5/8
Management of waste	4/4
Vector control	2/2
Equipment in examination rooms	2/4
Isolation measures	3/6
Patients with ARM	3/4
Hand hygiene	4/8
Personal hygiene	10/12
Protection of employees	5/8
Protective clothing	5/6
Medication	5/6
Use of antimicrobials	2/4
Miscellaneous	3/8
Total audit score/maximum IPC score (%)	59/102 (58%)

Abbreviations: IPC, infection prevention and control; ARM, antimicrobial resistant microorganisms.

## Data Availability

Data is available upon reasonable request.

## Supplementary Materials

The following supporting information can be downloaded at: https://www.mdpi.com/article/10.3390/antibiotics11020213/s1, Table S1: Antimicrobial resistant microorganisms isolated from the clinical environment; Table S2: Sequencing data for strains isolated in this study; Table S3: Co-location of antimicrobial resistance genes identified in hybrid assemblies of three selected carbapenem-producing isolates; Table S4: Criteria applied for the audit scoring; Table S5: List of environmental sampling sites in the small animal clinic.
