# Supplementary material for: Massive Spread of OXA-48 Carbapenemase-Producing Enterobacteriaceae in the Environment of a Swiss Companion Animal Clinic
Source: Antibiotics (Basel). 2022 Feb 8;11(2):213. doi: 10.3390/antibiotics11020213 (PMC8868282; doi:10.3390/antibiotics11020213)
Supplement: Supplementary file 1 [file antibiotics-11-00213-s001.zip › antibiotics-1557375-supplementary.pdf]

antibiotics

MDPI

Article

Massive Spread of OXA-48 Carbapenemase-Producing Enterobacteriaceae in the Environment of a Swiss Companion Animal Clinic

Kira Schmitt [1,2], Michael Biggel [1], Roger Stephan [1,*] and Barbara Willi [3]

[1] Institute for Food Safety and Hygiene, University of Zurich, CH-8057 Zurich, Switzerland; kira.schmitt@uzh.ch (K.S.); michael.biggel@uzh.ch (M.B.)
[2] Graduate School for Cellular and Biomedical Sciences, University of Bern, CH-3012 Bern, Switzerland
[3] Clinic for Small Animal Internal Medicine, University of Zurich, CH-8057 Zurich, Switzerland; bwilli@vetclinics.uzh.ch
* Correspondence: roger.stephan@uzh.ch; Tel.: +41-44-635-86-51

Supplementary Materials

Tables S1–S5 are available as Supplementary data and can be accessed online

Table S1. Antimicrobial resistant microorganisms isolated from the clinical environment.

		AMR type			Species							Gene						Zone diameters [mm]																MIC		
Strain ID	Sampling day	CPE	ESBL-E	MRS	*Enterobacter* spp.	*E. coli*	*K. pneumoniae*	*Serratia marcescens*	*Citrobacter braakii*	*Staphylococcus* spp.	MLST (CC)	OXA-48	CTX-M-15	SHV-1	SHV-12	TEM-1	MecA	AM	CZ	CTX	AMC	FEP	NA	CIP	SXT	FOS	AZM	F/M	S	K	GM	C	TE	Ertapenem	Meropenem	Imipenem
MV f2 C	2	■			■							■						6	6	18	6	19	6	8	6	18	9	16	7	6	14	6	6	1.5	0.38	0.38
MV f4 C	4	■			■							■						6	6	19	6	29	6	10	6	23	9	17	7	6	15	6	6	1.5	0.38	0.38
MV f4 O	4	■			■							■						6	6	18	6	22	6	9	6	21	9	17	8	6	14	6	6	1.5	0.38	0.38
MV hh1 O	1	■			■							■						6	6	19	6	21	6	6	6	23	9	19	6	6	17	6	6	1.5	0.38	0.25
MV hh1 SK1 C	1	■			■							■						6	6	16	6	18	6	6	6	15	8	15	8	6	15	6	6	2	0.38	0.38
MV hh1 SK2 C	1	■			■							■						6	6	20	6	24	6	10	6	25	10	17	7	6	16	6	6	2	0.38	0.38
MV kk2 C	2	■			■							■						6	6	13	6	23	6	10	6	20	10	19	8	6	16	6	6	4	2	1.5
MV kk2 O	2	■			■							■						6	6	7	6	27	6	10	6	14	11	15	7	6	14	6	6	1.5	0.25	0.38
MV l2 C	2	■			■							■						6	6	19	6	23	6	9	6	19	10	12	7	6	17	6	6	3	0.75	1
MV l2 O	2	■			■							■						6	6	19	6	24	6	8	6	21	9	16	7	6	14	6	6	2	0.25	0.38
MV n4 O	4	■			■							■						6	6	9	7	18	16	18	6	24	14	10	6	16	9	6	6	0.5	0.19	0.25
MV oo2 C	2	■			■							■						6	6	19	6	26	6	10	6	23	10	15	7	6	16	6	6	1.5	0.38	0.5
MV oo2 O	2	■			■							■						6	8	25	7	31	20	23	22	19	14	21	15	20	19	20	20	0.5	0.25	0.38
MV oo4 C *	4	■			■						ST114	■						6	6	20	6	27	6	10	6	24	10	16	7	6	16	6	6	2	0.25	0.38
MV oo4 O	4	■			■							■						6	6	8	7	18	15	18	6	22	12	10	6	13	8	6	6	0.5	0.25	0.38
MV r1 C *	1	■			■						ST114	■						6	6	23	6	20	6	8	6	24	8	15	7	7	15	6	6	2	0.38	0.38
MV r2 O	2	■			■							■						6	6	20	6	27	6	10	6	26	12	17	8	6	17	6	6	2	0.38	0.38
MV rr3 C	3	■			■							■						6	6	9	6	17	15	17	6	22	12	13	6	13	8	6	6	0.38	0.125	0.25
MV tt3 SK1 C	3	■			■							■						6	6	8	6	17	16	18	6	23	12	12	6	15	10	6	6	0.38	0.125	0.25
MV u2 C	2	■			■							■						6	6	22	6	28	6	10	6	27	12	18	8	6	16	6	6	3	0.38	0.38
MV u2 O	2	■			■							■						6	6	19	6	26	6	10	6	23	10	15	8	6	16	6	6	2	0.38	0.38
MV v2 C	2	■			■							■						6	6	20	6	27	6	10	6	25	10	16	8	6	17	6	6	2	0.38	0.75
MV v2 SK1 O	2	■			■							■						6	6	15	6	22	6	8	6	22	8	14	7	6	14	6	6	2	0.38	0.38
MV x1 C *	1	■			■						ST114	■						6	6	24	6	24	6	10	6	24	10	17	7	6	16	6	6	1.5	0.38	0.5
MV x1 SK2 O	1	■			■							■						6	6	20	6	26	6	10	6	24	10	16	7	6	17	6	6	1	0.25	0.38
MV xx2 C	2	■			■							■						6	6	21	6	27	6	10	6	22	11	15	8	6	16	6	6	2	0.38	0.38
MV xx2 O *	2	■			■						ST113	■						6	6	26	6	32	22	28	29	20	12	12	16	21	20	18	18	0.38	0.125	0.25
MV zz2 C	2	■			■							■						6	6	17	6	22	6	8	6	24	11	17	8	6	14	6	6	1.5	0.38	0.38
MV zz2 SK3 O	2	■			■							■						6	6	18	6	23	6	8	6	23	10	16	7	6	18	6	6	1.5	0.38	0.38
MV r4 SK2 C *	4	■				■					ST961 (ST12 Cplx)	■						6	8	24	6	28	26	28	28	30	15	22	13	14	20	20	18	0.25	0.19	0.25
MV c4 SK1 O	4	■				■					ST12 (ST12 Cplx)	■						6	8	23	6	29	23	24	24	28	18	23	16	18	20	19	19	0.5	0.25	0.38
MV v4 SK1 O	4	■				■					ST641 (ST86 Cplx)	■						6	7	20	6	21	21	24	22	29	14	19	14	13	15	20	19	0.5	0.25	0.25
MV zz2 SK2 O *	2	■				■					ST1406	■						6	11	21	6	25	25	24	25	28	14	19	14	18	18	19	18	0.5	0.38	0.25
MV u4 C	4	■					■				ST219	■						6	6	9	6	21	26	25	6	25	12	21	6	24	26	6	6	1.5	0.5	0.5
MV u1 SK2 O *	1	■					■				ST219	■						6	6	11	6	20	22	23	6	18	10	14	6	18	18	6	7	0.25	0.19	0.38
MV r1 SK2 O	1	■					■				ST219	■						6	6	12	6	18	24	23	6	22	9	18	6	21	22	6	7	0.25	0.19	0.25
MV u4 SK1 O	4	■					■				ST219	■						6	6	11	7	21	26	25	6	23	8	14	6	22	20	6	7	0.25	0.19	0.5
MV x1 SK1 O	1	■					■				ST5580	■						6	15	27	6	28	18	25	23	25	12	19	18	23	22	21	16	0.5	0.25	0.25
MV v4 SK2 O *	4	■					■				ST5873	■						6	12	22	6	23	19	23	22	20	12	17	14	18	16	21	20	0.38	0.19	0.25
MV c4 C	4	■					■				ST3063	■						6	6	20	6	21	24	22	22	19	15	9	15	18	16	18	17	8	6	32
MV c4 SK2 O *	4	■					■				ST3063	■						6	11	23	6	24	21	22	23	22	12	16	14	18	17	19	20	0.38	0.19	0.25
MV zz2 SK1 O	2	■					■				ST3063	■						6	14	27	7	30	23	28	26	25	16	15	18	23	21	25	23	0.25	0.125	0.25
MV u4 SK2 O *	4	■					■				ST219	■						6	6	12	6	20	22	21	6	20	8	18	6	21	20	24	7	0.38	0.25	0.25
MV u1 SK1 O *	1	■						■				■						6	6	25	6	28	22	20	6	28	9	8	6	22	20	20	6	0.5	0.38	1
MV v1 O	1	■						■				■						6	6	20	6	32	28	30	30	27	12	9	19	24	23	21	7	0.94	0.94	0.75
MV u1 SK2 C	1	■						■				■						6	6	19	6	24	22	17	6	26	10	8	7	20	18	19	6	1	0.75	1.5
MV v2 SK2 O	2	■						■				■						6	6	24	6	30	25	27	22	26	10	8	17	20	21	18	7	0.5	0.125	1
MV f2 SK1 O	2	■						■				■						6	6	22	6	25	23	24	24	26	12	6	16	21	19	22	9	0.25	0.19	0.75
MV r1 SK3 O *	1	■							■			■						6	9	25	6	31	17	24	6	33	13	25	10	22	20	21	6	0.25	0.19	0.25
MV u1 SK3 O	1	■							■			■						6	8	22	6	24	16	21	6	28	15	21	9	19	19	20	6	0.25	0.19	0.25
MV u4 SK3 O	4	■							■			■						6	8	23	6	25	16	19	6	26	12	23	10	18	17	18	6	0.38	0.19	0.25
MV r1 SK1 O	1	■							■			■						6	7	24	6	28	17	23	6	29	15	22	9	20	19	22	6	0.25	0.19	0.38
MV r2 SK2 E	2		■		■													6	6	19	6	26	6	9	6	27	10	16	9	6	17	6	6	n.a.		
MV u2 E	2		■		■										■			6	12	25	23	32	18	25	6	36	16	22	6	10	6	6	6	n.a.		
MV hh1 SK1 E	1		■		■													6	6	16	6	22	6	7	6	23	8	14	7	6	14	6	6	n.a.		
MV hh1 SK3 E	1		■		■													6	6	13	6	19	6	7	6	20	8	11	7	6	13	6	6	n.a.		
MV x1 E	1		■		■								■			■		6	6	6	6	14	6	6	6	21	13	13	6	13	8	6	7	n.a.		
MV zz2 E	2		■		■										■			6	8	22	17	24	21	25	6	30	12	17	6	8	6	6	6	n.a.		
MV hh1 SK4 E	1		■		■													6	6	16	6	20	6	8	6	21	8	14	7	6	13	6	6	n.a.		
MV hh1 SK2 E	1		■		■													6	6	7	6	20	6	8	6	21	8	15	7	6	14	6	6	n.a.		
MV r2 SK1 E	2		■		■										■			11	14	30	9	34	26	32	30	28	12	13	16	21	20	19	18	n.a.		
MV oo4 SK1 E	4		■		■								■			■		6	6	8	6	16	14	16	6	20	13	13	6	12	9	6	6	n.a.		
MV n4 E	4		■		■								■			■		6	6	9	6	16	14	17	6	22	12	14	6	13	9	6	6	n.a.		
MV f4 SK1 E	4		■		■													6	6	16	6	23	6	7	6	26	9	16	7	6	13	6	6	n.a.		
MV f4 SK2 E	4		■		■													6	6	15	6	24	6	10	6	28	10	17	8	6	15	6	6	n.a.		
MV r4 E	4		■		■								■			■		6	6	9	6	14	11	20	6	15	13	12	6	13	9	6	6	n.a.		
MV l2 E	2		■		■													6	6	17	6	26	6	8	6	20	10	15	9	6	14	6	6	n.a.		
MV aa3 SK2 E	3		■		■										■			6	10	20	21	28	22	30	6	32	12	17	6	8	6	6	6	n.a.		
MV kk2 SK2 E	2		■		■													6	6	10	6	25	6	8	6	10	10	15	7	6	14	6	6	n.a.		
MV kk2 SK1 E	2		■		■													6	6	10	6	15	6	8	6	14	10	18	6	6	14	6	6	n.a.		
MV aa3 SK3 E	3		■		■										■			6	12	24	26	30	24	32	6	31	12	18	6	9	6	6	6	n.a.		
MV rr3 E	3		■		■								■			■		6	6	8	6	18	15	17	6	23	12	12	6	12	8	6	6	n.a.		
MV oo4 SK2 E	4		■		■										■	■		6	6	12	7	23	6	6	6	24	7	15	6	8	8	19	6	n.a.		
MV oo2 E	2		■				■				ST219		■	■				6	6	8	6	15	11	19	6	19	8	14	6	18	19	21	8	n.a.		
MV r1 E	1		■				■				ST219		■	■				6	6	10	6	18	20	22	6	18	8	13	6	16	18	6	6	n.a.		
MV f2 E	2		■				■				ST219		■	■				6	6	9	18	18	22	18	6	15	8	14	6	19	18	6	7	n.a.		
MV cc3 E	3		■				■				ST219		■	■				6	6	11	22	18	21	24	6	23	9	14	6	21	19	6	7	n.a.		
MV u4 E	4		■				■				ST219		■	■				6	6	12	6	18	22	22	6	19	8	17	6	22	20	6	7	n.a.		
MV q1 M	1			■						■							■	n.a.																n.a.		
MV ff1 M	1			■						■							■	n.a.																n.a.		
MV c1 M	1			■						■							■	n.a.																n.a.		
MV aa1 M	1			■						■							■	n.a.																n.a.		
MV ee3 M	3			■						■							■	n.a.																n.a.		
MV cc3 M	3			■						■							■	n.a.																n.a.		
MV a4 M	4			■						■							■	n.a.																n.a.		
MV 7bp M	4			■						■							■	n.a.																n.a.		
MV 7ap M	4			■						■							■	n.a.																n.a.		

Abbreviations: ESBL-E, extended-spectrum beta-lactamase producing Enterobacteriaceae; CPE, carbapenemase-producing Enterobacteriaceae; MRS, methicillin-resistant staphylococci; MLST, multi-locus sequence typing; CC, clonal complex; AM, ampicillin; CZ, cephazolin; CTX, cefotaxime; AMC, amoxicillin-clavulanic acid; FEP, cephepim; NA, nalidixic acid; CIP, ciprofloxacin; SXT, sulfamethoxazole trimethoprim; FOS, fosfomycin; AZM, azithromycin; F/M, nitrofurantoin; S, streptomycin; K kanamycin; GM, gentamicin; C, chloramphenicol; TE, tetracycline; MIC, minimal inhibitory concentration (mg/ml). *, whole genome sequencing conducted

Table S2. Sequencing data for strains isolated in this study.

Strain	Assembly level	Biosample	Genome accession number	Species	MLST scheme	MLST	Resistance genes
MV-r1-SK3-O	draft	SAMN22551378	JAJGOG000000000	*Citrobacter braakii*	*Citrobacter freundii*	-	*aadA2*, *bla*CMY-101, *bla*OXA-48, *bla*TEM-1B, *dfrA16*, *ere*(A), *qac*Edelta1, *qnr*B51, *qnr*S1, *sul*1, *tet*(D)
MV-oo4-C	draft	SAMN22551379	JAJGOF000000000	*Enterobacter hormaechei*	*Enterobacter cloacae complex*	114	*aadA2*, *ant(2'')-Ia*, *ant(3'')-Ia*, *aph(3'')-Ib*, *aph(3')-Ia*, *aph(6)-Id*, *bla*ACT-16, *bla*OXA-48, *catA2*, *dfrA1*, floR, *fos*A, *mcr*-9, *qac*Edelta1, *qnr*A1, *sul*1, *tet*(D)
MV-r1-C	draft	SAMN22551380	JAJGOE000000000	*Enterobacter hormaechei*	*Enterobacter cloacae complex*	114	*aadA2*, *ant(2'')-Ia*, *ant(3'')-Ia*, *aph(3'')-Ib*, *aph(3')-Ia*, *aph(6)-Id*, *bla*ACT-16, *bla*OXA-48, *catA2*, *dfrA1*, *flo*R, *fos*A, *mcr*-9, *qac*Edelta1, *qnr*A1, *sul*1, tet(D)
MV-x1-C	draft	SAMN22551381	JAJGOD000000000	*Enterobacter hormaechei*	*Enterobacter cloacae complex*	114	*aadA2*, *ant(2'')-Ia*, *ant(3'')-Ia*, *aph(3'')-Ib*, *aph(3')-Ia*, *aph(6)-Id*, *bla*ACT-16, *bla*OXA-48, *cat*A2, *dfrA1*, *flo*R, *fos*A, *mcr*-9, *qac*Edelta1, *qnr*A1, *sul*1, *tet*(D)
MV-xx2-O	draft	SAMN22551382	JAJGOC000000000	*Enterobacter hormaechei*	*Enterobacter cloacae complex*	113	*bla*ACT-15, *bla*OXA-48, *fos*A
MV-r4-SK2-C	draft	SAMN22551383	JAJGOB000000000	*Escherichia coli*	*Escherichia coli*	961	*bla*OXA-48
MV-zz2-SK2-O	draft	SAMN22551384	JAJGOA000000000	*Escherichia coli*	*Escherichia coli*	1406	*bla*OXA-48
MV-c4-SK2-O	draft	SAMN22551385	JAJGNZ000000000	*Klebsiella pneumoniae*	*Klebsiella pneumoniae*	3063	*oqx*A, *oqx*B, *bla*OXA-48, *bla*SHV-65, *fos*A5

MV-u1-SK2-O	complete (hybrid assembly)	SAMN22551386	CP085866-CP085870	*Klebsiella pneumoniae*	*Klebsiella pneumoniae*	219	*oqxA*, *oqxB*, *aad*A2, *aph(3'')-Ib*, *aph(6)-Id*, *bla*CTX-M-15, *bla*OXA-48, *bla*SHV-145, *catA2*, *dfr*A12, *fos*A, *mph*(A), *qac*Edelta1, *qnr*S1, *sul*1, *sul*2, *tet*(A)
MV-u4-SK2-O	draft	SAMN22551387	JAJGNY000000000	*Klebsiella pneumoniae*	*Klebsiella pneumoniae*	219	*oqx*A, *oqx*B, *aad*A2, *aph(3'')-Ib*, *aph(6)-Id*, *bla*CTX-M-15, *bla*OXA-48, *bla*SHV-145, *dfrA*12, *fos*A, *mph*(A), *qac*Edelta1, *qnr*S1, *sul*1, *sul*2, *tet*(A)
MV-v4-SK2-O	complete (hybrid assembly)	SAMN22551388	CP085863-CP085865	*Klebsiella pneumoniae*	*Klebsiella pneumoniae*	5873	*oqx*A, *oqx*B, *bla*OXA-48, *bla*SHV-187, *fos*A
MV-u1-SK1-O	complete (hybrid assembly)	SAMN22551389	CP085860-CP085862	*Serratia marcescens*	-	-	*aac(6')-Ic*, *aadA2*, *bla*OXA-48, *bla*SRT-2, *bla*TEM-1B, *dfrA*16, *ere*(A), *qac*Edelta1, *qnr*S1, *sul*1, *tet*(41), *tet*(D)

Table S3. Co-location of antimicrobial resistance genes identified in hybrid assemblies of three selected carbapenem-producing isolates.

Isolate	Accession	Element	Size (bp)	Resistance genes
***Serratia marcescens* MV-u1-SK1-O**	CP085860	Chromosome	5188985	*bla*SRT-2, *tet(41)*, *aac(6′)-Ic*
	CP085861	Plasmid pOXA48_MV-u1-SK1-O-a	63589	*bla*OXA-48
	CP085862	Plasmid pMV-u1-SK1-O-b	57983	*tet(D)*, *dfrA16*, *aadA2*, *ere(A)*, *qac*Edelta1, *sul1*, *bla*TEM-1B, *qnrS1*
***Klebsiella pneumoniae* MV-v4-SK2-O**	CP085863	Chromosome	5263198	*oqxB*, *oqxA*, *bla*SHV-187, *fosA*
	CP085864	Plasmid pOXA48_MV-v4-SK2-O-a	63589	*bla*OXA-48
	CP085865	Plasmid pMV-v4-SK2-O-b	5891	
***Klebsiella pneumoniae* MV-u1-SK2-O**	CP085866	Chromosome	5320804	*oqxB*, *oqxA*, *bla*CTX-M-15, *bla*SHV-145, *fosA*
	CP085867	Plasmid pMV-u1-SK2-O-a	202882	*tet(A)*, *mph(A)*, *sul1*, *qac*Edelta1, *aadA2*, *dfrA12*, *sul2*, *aph(3′′)-Ib*, *aph(6)-Id*, *qnrS1*, *catA2*
	CP085868	Plasmid pOXA48_MV-u1-SK2-O-b	63589	*bla*OXA-48
	CP085869	Plasmid pMV-u1-SK2-O-c	4667	
	CP085870	Plasmid pMV-u1-SK2-O-d	4060	

Table S4. Criteria applied for the audit scoring. The scoring system for the infection prevention and control (IPC) audits has been adapted from previous publications [1,2]. Each IPC category was scored as follows: criteria fulfilled= score 2; criteria partially fulfilled=score 1; criteria not fulfilled= score 0. A score per IPC area and a total score was calculated (maximum total score= 102). The results of the IPC scoring are shown in Table 1.

Area of IPC	Topic	Criteria for assessment	Not implemented	Partially implemented	Fully implemented	Comments
**IPC management**	Hygiene manual	Written IPC manual containing the relevant areas of IPC and provided in written/online form to all employees.				
	Designated IPC team/ person	A defined person or group of people (in large clinics) responsible for IPC implementation, control and teaching that has the according knowledge; in case of IPC groups: regular meetings take place.				
	Staff involvement	Staff involved in IPC.				
	Regular audits/ Surveillance	Regular IPC audits in the clinic/practice by the IPC mandatory/team or an external IPC advisor, taking place on a regular base. Assessment of staff compliance and knowledge of IPC measures.				
	Reportable diseases	List of all reportable diseases and contact numbers for veterinary and public health authorities available.				
**Staff education**	Frequency	Documented annual education on IPC and and protective practices. New staff and volunteers trained and provided with information on IPC practice.				
	Hand hygiene	Education on how and when to use hand hygiene for all employees involved in clinical service, hold on a regular base.				
	Protective practices	Education provided regarding protective practices.				
	General IPC	Education in infection prevention and control for all employees involved in clinical service, hold on a regular base.				
	Antimicrobial stewardship	Education in antimicrobial stewardship for all employees involved in prescription and application of antimicrobials, hold on a regular base.				

	Early identification of infectious patients	All staff trained to identify potentially infectious patients when making appointments. Determining if infection control practices are necessary before animal enters practice. List of symptoms of potentially infectious diseases available to receptionists.
**Cleaning/ disinfection**	Written & updated protocols	Disinfection protocols with type of disinfection, concentrations and residence times defined for different areas/equipment; written checklists to confirm regular cleaning/disinfection in different areas.
	Spectrum and application	Licensed disinfection compounds for clinical use with sufficient bactericidal and virucidal spectrum and use in accordance with the manufacturer's instructions; cleaning procedures that allow for removal of organic matter and proper disinfection.
	Autoclave	Quality control sterility indicators used in autoclaved pack. Biological indicators periodically used to ensure adequate sterilization. Results recorded.
	Information dissemination	Written protocols in all areas and available to all people involved in cleaning/disinfection. Safety data sheets for disinfectant products available.
**Waste**	Sharps	Approved puncture-resistant, labeled containers used. Containers not more than ¾ filled. Containers accessible in all required areas. Sharps disposed immediately after use.
	Biohazardous and non-biohazardous	Clear guidelines available.
**Vector control**	Rodent control	Food debris and clutter eliminated. Entry points closed. No standing water on site. Windows screened.
**Examination rooms**	Hand washing stations	Hand washing stations in all examination rooms.
	Supplies	Only essential supplies present.
**Isolation measures**	Structure and work-flow	Patients with potential contagious diseases physically separated from other patients; isolation ward that allows for adequate patient separation that contains an upstream area with provision of hand sanitizer and protective clothing; utensils and equipment are assigned to each patient and remain in the isolation area until final cleaning/disinfection;

		no additional material stored within the isolation room.
	Information dissemination	Diseases requiring specific isolation/protective measures are specified and the information is available in written form to all employees.
	Cleaning/ disinfection	Disinfectants also covering parvovirus and fungal spores are used in the isolation areas; cleaning/disinfection procedures are defined and available to all employees. Footbaths or footmats available.
**Guidelines for patients with ARM**	Definition/ designation of ARM patients	ARM are defined and patients with ARM infections are designated.
	Protective measures	ARM-infected patients are physically separated from other patients, patient movement in the institution is restricted, disinfection procedures are defined.
**Hand hygiene**	Hand sanitizer	Hand sanitizer in single-use containers and dispensers are available at all hand washing stations; hand washing stations in all consultation and treatment rooms, wards, laboratories and toilets.
	Washing lotion	Medical hand washing lotion in single-use containers available at all hand washing stations (see above).
	Skin protection products	Presence of skin care products available at all hand washing stations (see above).
	Disposable towels	Use of single-use disposable towels at all hand washing stations (see above).
**Personal hygiene**	Working clothes	Provision of working clothes (trousers, and top or coat) for all employees.
	Hand jewelry and nails	No hand jewelry (watches, rings, bracelets or similar) and no long or artificial nails and nail polish allowed for employees involved in clinical work.
	Food consumption	No consumption of food by employees in the patient areas.
	Food storage	No food of employees stored within the patient areas/refrigerators.
	Personnel changing rooms	Changing rooms available for all employees that allow to separate private and working clothes.

	Laundry	Daily change of working clothes specified; working clothes are cleaned by an external company or by an in-house washing machine in the clinic. Laundry dried at high temperatures (65-70°C). Infectious laundry presoaked in bleach solution. Soiled laundry transported in a clean manner. Clean laundry separated from soiled laundry. Hand hygiene available in laundry area.
**Protection of employees**	Vaccinations	Tetanus and rabies vaccination recommended for all employees and costs covered by the clinic/practice.
	Measures for pregnant/ immunosuppressed employees	Protective measures in case of pregnancy and/or immunosuppression defined and communicated to all employees.
	Zoonoses	Written protocols for management of potential staff exposure to zoonotic pathogens.
	Animal bite	Animal bite policy in place.
**Protective clothing**	Composition	Complete protective equipment including disposable protective coats or overalls, gloves, shoe covers, eye protection and masks (surgical, N95 mask).
	Use	Situation requiring protective clothing and correct use of protective clothing specified and information provided in written form to all employees involved in clinical work.
	Storage and provision	Protective clothing provided in front of the isolation area and with low potential for contamination of the equipment.
**Medication**	Preparation	Preparation of medication on a clean and disinfected working area; avoidance of preparation of medication in advance. Hygiene measures when preparing medication.
	Storage	Storage according to official regulations (e.g. temperature, storage time); in a clean area/fridge; with no contact to food, feed or patient samples.
	Dating of open vials	Dating of open vials and consistently done for all medications.
**Use of antimicrobials**	Guidelines on antimicrobial use and dosing	Specified guidelines on use and dosing of antimicrobials available for all employees involved in prescription and application of antimicrobials.

	Restrictions for critically important antimicrobials	Limited or prohibited use of antibiotics of last resort, restrictions are communicated and known to all employees.
**Miscellaneous**	Diagnostic specimen	Designated area for handling diagnostic specimens.
	Raw meet	Raw meet feeding policy for hospitalized animals.
	Printed materials for clients	Printed materials for clients on zoonotic diseases available.
	Policy for shelter animals	Policy regarding the admittance of shelter/homeless animals.

Abbreviations: IPC, Infection prevention and control; **ARM**, antimicrobial resistant organisms.

Table S5. List of environmental sampling sites in the small animal clinic.

Identification code	Surface
MV_a	Oxygen device
MV_b	Mobile phone
MV_c	Keyboard
MV_d	Computer mouse
MV_e	Treatment table
MV_f	Water tap
MV_g	Drawer
MV_h	Large cabinet
MV_i	Small cabinet
MV_j	Drug preparation area
MV_k	Small boxes on medication preparation surface
MV_l	Refrigerator
MV_m	Infusion pump
MV_n	Thermometer
MV_o	Stethoscope
MV_p	Soap dispenser
MV_q	Alcohol dispenser
MV_r	Cat cage
MV_s	Dog cage
MV_t	Blood pressure monitor
MV_u	Sewer gutter
MV_v	Floor
MV_w	Floor of the scale
MV_x	Display of the scale
MV_y	Scissors
MV_z	Clippers
MV_aa	Othoscope
MV_bb	Anesthesia machine
MV_cc	Toilet door handle
MV_dd	Toilet flush
MV_ee	Bowl
MV_ff	Door
MV_gg	Medication
MV_hh	Washing machine
MV_jj	Gurney
MV_kk	Heating device
MV_mm	Locker
MV_nn	Cleaning spray
MV_oo	Patient table with sink
MV_pp	Medical dishwasher
MV_qq	Coffee machine
MV_rr	Ultrasound machine
MV_tt	Microwave
MV_uu	X-ray machine
MV_vv	Medicine
MV_ww	Heat sealing device
MV_xx	SpO2 device

MV_yy	Transportation box
MV_zz	Centrifuge
MV_aaa	Operation table lamp

References

1. Schmidt, J.S.; Kuster, S.P.; Nigg, A.; Dazio, V.; Brilhante, M.; Rohrbach, H.; Bernasconi, O.J.; Büdel, T.; Campos-Madueno, E.I.; Gobeli Brawand, S.; et al. Poor infection prevention and control standards are associated with environmental contamination with carbapenemase-producing Enterobacterales and other multidrug-resistant bacteria in Swiss companion animal clinics. *Antimicrob. Resist. Infect. Control* **2020**, *9*, 93.
2. Stull, J.W.; Bjorvik, E.; Bub, J.; Dvorak, G.; Petersen, C.; Troyer, H.L. 2018 AAHA Infection Control, Prevention, and Biosecurity Guidelines. *J. Am. Anim. Hosp. Assoc.* **2018**, *54*, 297–326, doi:10.5326/JAAHA-MS-6903.
